# Multidisciplinary and participatory workshops with stakeholders in a community of extreme poverty in the Peruvian Amazon: Development of priority concerns and potential health, nutrition and education interventions

**DOI:** 10.1186/1475-9276-6-6

**Published:** 2007-07-10

**Authors:** Martin Casapia, Serene A Joseph, Theresa W Gyorkos

**Affiliations:** 1 Asociación Civil Selva Amazónica, Iquitos, Peru; 2 Division of Clinical Epidemiology, McGill University Health Centre, 687 Pine Avenue West, Pavilion V, Montreal, Quebec, H3A 1A1, Canada; 3 Department of Epidemiology, Biostatistics and Occupational Health, McGill University, Montreal, Quebec, Canada

## Abstract

**Background:**

Communities of extreme poverty suffer disproportionately from a wide range of adverse outcomes, but are often neglected or underserved by organized services and research attention. In order to target the first Millennium Development Goal of eradicating extreme poverty, thereby reducing health inequalities, participatory research in these communities is needed. Therefore, the purpose of this study was to determine the priority problems and respective potential cost-effective interventions in Belen, a community of extreme poverty in the Peruvian Amazon, using a multidisciplinary and participatory focus.

**Methods:**

Two multidisciplinary and participatory workshops were conducted with important stakeholders from government, non-government and community organizations, national institutes and academic institutions. In Workshop 1, participants prioritized the main health and health-related problems in the community of Belen. Problem trees were developed to show perceived causes and effects for the top six problems. In Workshop 2, following presentations describing data from recently completed field research in school and household populations of Belen, participants listed potential interventions for the priority problems, including associated barriers, enabling factors, costs and benefits.

**Results:**

The top ten priority problems in Belen were identified as: 1) infant malnutrition; 2) adolescent pregnancy; 3) diarrhoea; 4) anaemia; 5) parasites; 6) lack of basic sanitation; 7) low level of education; 8) sexually transmitted diseases; 9) domestic violence; and 10) delayed school entry. Causes and effects for the top six problems, proposed interventions, and factors relating to the implementation of interventions were multidisciplinary in nature and included health, nutrition, education, social and environmental issues.

**Conclusion:**

The two workshops provided valuable insight into the main health and health-related problems facing the community of Belen. The participatory focus of the workshops ensured the active involvement of important stakeholders from Belen. Based on the results of the workshops, effective and essential interventions are now being planned which will contribute to reducing health inequalities in the community.

## Background

Poverty is the main underlying cause of ill health in many rural and peri-urban communities in Africa, Asia and Latin America. Many of the poorest communities lack the most basic of human needs. Most are also neglected by any organized local government service, non-governmental agency or respective international counterparts. It has been estimated that one in every four persons who live in developing countries, or approximately 1.2 billion people, live in conditions of extreme poverty (*i.e. *income of less than 1 US dollar per day) [1].

In September 2000, at the Millennium Summit, eight Millennium Development Goals (MDGs) were identified to address the plight of the world's poor [2]. These included goals related to health, education, gender and the environment. The first MDG focuses on eradicating extreme poverty and hunger, and to achieve 50% of this goal by 2015. Evidence to date clearly establishes the links between poverty and personal, community and system-based shortcomings in health, nutrition and education domains [3,4]. It is therefore essential to target those vulnerable populations living in communities of extreme poverty as they suffer disproportionately from a wide-range of adverse outcomes.

According to the most recent statistics, Latin America has currently met or is on track to meet several Millennium Development Goals [5]. However, there has been minimal improvement in reducing poverty, attributed mainly to poor economic growth in the region, and most countries are unlikely to meet the expected target. In Peru, for example, the proportion of those living below 1 US dollar per day increased from 9.4 % to 18.1 % from 1994 to 2000 [6].

Like many Latin American countries, Peru is faced with significant inequalities within its population, leading to vulnerable sectors that are not accurately represented in national statistics [5]. This includes those that live in remote and rural areas as well as in areas of extreme poverty. One such community is Belen (latitude: 3°45'54.20"S, longitude: 73°14'49.17"W [7]) in the Peruvian Amazon. Belen is located outside of Iquitos, the capital city of Loreto which is the largest region of the Peruvian Amazon [8]. Due to Belen's location on the banks of the Itaya/Amazon River and its propensity for seasonal flooding, the houses are constructed on wooden stilts or on floating platforms. Of the over 65,000 inhabitants of Belen [9], most do not have access to reliable potable water for drinking or improved sanitation systems. Human waste contaminates the water directly from floating latrines or is removed by natural elements from primitive land-based latrines leading to a state of permanent faecal contamination. Most residents of Belen make their livelihood by selling local produce and fish in the daily market.

Due to the high levels of poverty, difficulties in access to care, and local beliefs and customs, many in this population do not seek conventional medical services until late in an illness and do not attend health centres during a pregnancy or at delivery. This can lead to increasing transmission and complications of prevalent illnesses. However, accurate statistics have generally not been available because there has been little interest and no resources to carry out a proper study in this community. Previous research that was conducted in the area showed a higher prevalence of infectious and parasitic infections in pregnant women in Belen compared to other neighbouring areas [unpublished], and therefore suggested a need for improved understanding of the health and health-related concerns of this population.

To better understand the main health and social concerns in Belen, two multidisciplinary and participatory workshops were planned involving important stakeholders in the community. In addition, two surveys were planned to be conducted in the interim, in order to generate real-time data to inform the second workshop. The objective of the first workshop was to determine the priority problems in Belen, including associated causes and effects. The objective of the second workshop was to determine potential interventions to target the priority concerns in Belen, and to identify factors related to the implementation of the proposed interventions.

## Methods

This research was part of a project funded by a Global Health Research Pilot Project Grant of the Canadian Institutes of Health Research (CIHR) to obtain accurate statistics on several MDG indicators in Belen. This research was of a participatory focus involving stakeholders in Belen in all stages of the project from design to dissemination. The results of the school-based [10] and household-based [11] surveys have been published separately. The focus of this paper is to report on the workshops. Both workshops were conducted in Spanish. Ethics approvals were obtained from the McGill University Health Centre in Canada and the Ministry of Health in Peru.

### Workshop 1

The first multidisciplinary and participatory workshop was planned as a one day meeting at the beginning of the project (April 2005) prior to conducting the school and household surveys. This allowed for input from participants into survey design and implementation to improve the focus of the surveys. It was also an opportunity to inform the community about the surveys, thereby increasing community acceptance and participation. Invited participants included important stakeholders from a wide range of sectors including government and non-government organizations, community groups and national institutes. The workshop was divided into two sessions: 1) morning presentations by representatives of specific organizations to inform participants on the role of the various sectors and the needs of the community in Belen; and 2) afternoon participatory group work to identify priority problems and concerns of the Belen community.

The afternoon session was facilitated by the local project director and co-investigator of the project (MC). Participants were divided into small groups with representation from diverse sectors in each group. In step one, groups were asked to brainstorm as many problems that they felt were of concern in Belen. Once an exhaustive list was produced, groups then prioritized their list of problems using the following criteria: 1) frequency of the problem in the target population; 2) mortality impact of the problem on the target population; 3) potential feasibility of developing interventions for the problem; and 4) likely impact of potential interventions for the problem on the target population. These criteria were used to identify and focus resources on those problems that were of greatest impact on the population and that were modifiable through feasible and effective interventions in the community. For each problem, each criterion was given a score out of 5, with a maximum total score of 20. Problems were then ranked based on their scores to establish the top scoring problems in each group. Results of each group were discussed among all participants and a final consensus list of the top 10 priority problems was developed.

In step two, groups were provided with one of the top 6 priority problems to develop a cause and effect problem tree for the assigned problem based on the ZOPP Objectives-Oriented Project Planning approach [12]. Once completed, a representative from each group presented their results and participants from other groups were able to provide input into any modifications or additions to the problem tree.

### Workshop 2

The second multidisciplinary and participatory workshop took place following the completion of the school and household surveys (in March 2006). This allowed for real time data to inform past knowledge and perceptions discussed at the first workshop. The combination of these sources of qualitative and quantitative data from existing and new sources contributed to an improved knowledge base from which more accurate interventions could be identified in the second workshop. Invited participants included those that attended the first workshop as well as any other important stakeholders that were identified following the first workshop.

The workshop was divided into two sessions: 1) morning presentations by project co-investigators regarding a summary of the results of the two surveys conducted since the first workshop; and 2) afternoon participatory group work to develop potential interventions for the priority problems expressed at the first workshop. The afternoon sessions were again facilitated by the local project director (MC). Each group was assigned a priority problem from the first workshop and was asked to recommend potential interventions to address the problem, as well as possible costs and/or benefits of each intervention. Once interventions were determined, factors relating to the implementation of the interventions, including enabling factors and barriers to overcome, were identified. Results of the small group work were presented and discussed among all participants to provide additional comments and modifications.

## Results

### Global Health Research Pilot Project – Belen

**Figure 1:**
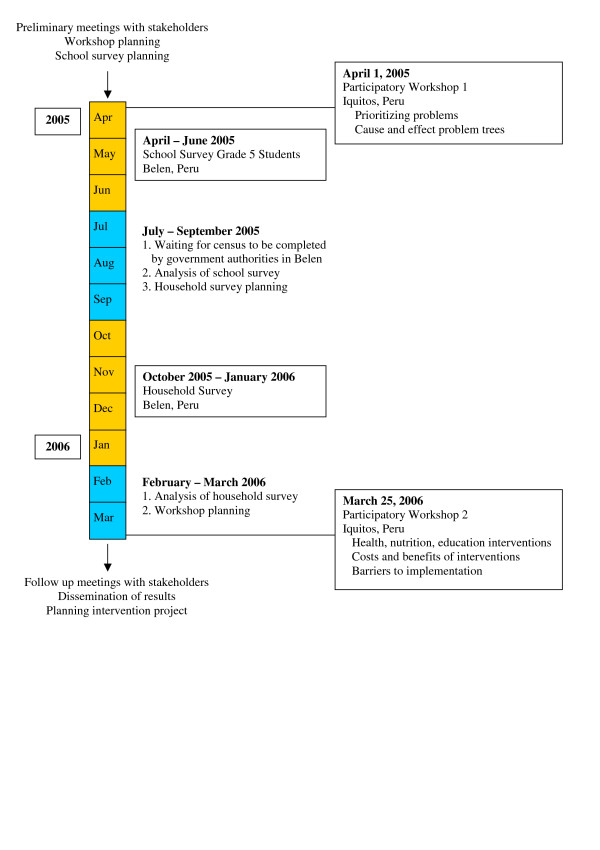
Phases of the Global Health Research Pilot Project in Belen, Peru, April 2005–March 2006.

The Global Health Research Pilot Project in Belen was conducted in four phases: 1) Participatory Workshop 1; 2) School survey in Grade 5 students in Belen; 3) Household survey of random sample of houses in Belen; and 4) Participatory Workshop 2 (Figure 1).

### Workshop 1

The first participatory workshop entitled "Taller Participativo 1: Un Enfoque Multidisciplinario para Reducir la Pobreza Extrema" (Participatory Workshop 1: A Multidisciplinary Approach to Reduce Extreme Poverty) was held at the El Dorado Plaza Hotel in Iquitos, Peru on April 1, 2005. Participants included representatives from: 1) Ministerio de Salud (Ministry of Health) including the Dirección Regional de Loreto (Regional Direction of Loreto), Comités Locales de Administración de Salud (CLAS) (Local Committees for Health Administration) and health centres; 2) Ministerio de Educación (Ministry of Education) including Dirección Regional de Loreto (Regional Direction of Loreto); 3) Ministerio de la Mujer y Desarrollo Social (Ministry of Women and Social Development) including the Wawa Wasi (Children's Homes) child day care program and the Programa Nacional de Asistencia Alimentaria (PRONAA) (National Program for Nutritional Assistance); 4) the Instituto Nacional de Estadística e Informática (National Institute of Statistics and Informatics); 5) the Municipality of Belen including the Mayor, the Defensoria Municipal del Niño y el Adolescente (DEMUNA) (Municipal Defence of the Child and Adolescent) and the Vaso de Leche (Glass of Milk) feeding program; 6) non-governmental associations (NGOs) representing community interests, including the Asociación Civil Selva Amazónica (Amazon Jungle Civil Organization), the Instituto de Investigación Nutricional (Nutritional Research Institute), Red Cross, Cáritas and Centro de Promoción Amazónica (CEPROA) (Centre for the Promotion of the Amazon); 7) community groups including the Junta Vecinal (neighbourhood association), the Asociación de Padres de Familia (Parents' Association) and community representatives in the municipality; and 8) universities including McGill University (Montreal, Canada), Universidad Peruana Cayetano Heredia (Lima, Peru) and the Universidad Nacional de la Amazonía Peruana (Iquitos, Peru). There were a total of 46 participants, of which 26 (56.5%) were women.

Morning presentations were given by representatives of the Ministry of Health, Ministry of Education, Ministry of Women and Social Development (Wawa Wasi), Nutritional Research Institute, the Municipality of Belen (Mayor, Vaso de Leche and DEMUNA), and the Asociación Civil Selva Amazónica.

The afternoon session involved small and large group work by participants.

For the small group work, there were six groups in total. After brainstorming and ranking all the problems based on the ranking criteria, the top five priority problems from each group were presented to all participants (Table 1).

**Table 1:** Priority problems ranked by frequency, mortality, interventions and impact from Participatory Workshop 1, Iquitos, Peru, April 1, 2005.

**Priority**	**Group 1**	**Group 2**	**Group 3**	**Group 4**	**Group 5**	**Group 6**
1	Environmental contamination; lack of values	Tuberculosis	Infant malnutrition	Personal insecurity; environmental contamination	Infant malnutrition	Infant malnutrition; childhood anaemia
2	Personal insecurity	Malnutrition	Diarrhoea	Overcrowding	STDs	Lack of basic sanitation
3	STDs	Diarrhoea; anaemia	Anaemia (children, pregnancy); lack of basic sanitation	Low school performance	Lack of basic sanitation; ARI; diarrhoea; skin diseases	Low education level
4	Low level of education; parasites; lack of community participation	Parasites	Parasites; lack of money for education	Lack of cultural identity	Domestic violence; adolescent pregnancy; lack of political will	Adolescent pregnancy
5	Childhood malnutrition; Inadequate parenting	Adolescent pregnancy	Alcohol/drug related violence and abuse; Prostitution			Unemployment

Based on a discussion of the results, a consensus was made on the top 10 priority problems in Belen. In order of priority, these were: 1) infant malnutrition; 2) adolescent pregnancy; 3) anaemia; 4) diarrhoea; 5) parasites; 6) lack of basic sanitation; 7) low level of education; 8) sexually transmitted diseases (STDs); 9) domestic violence; and 10) delayed school entry.

**Figure 2:**
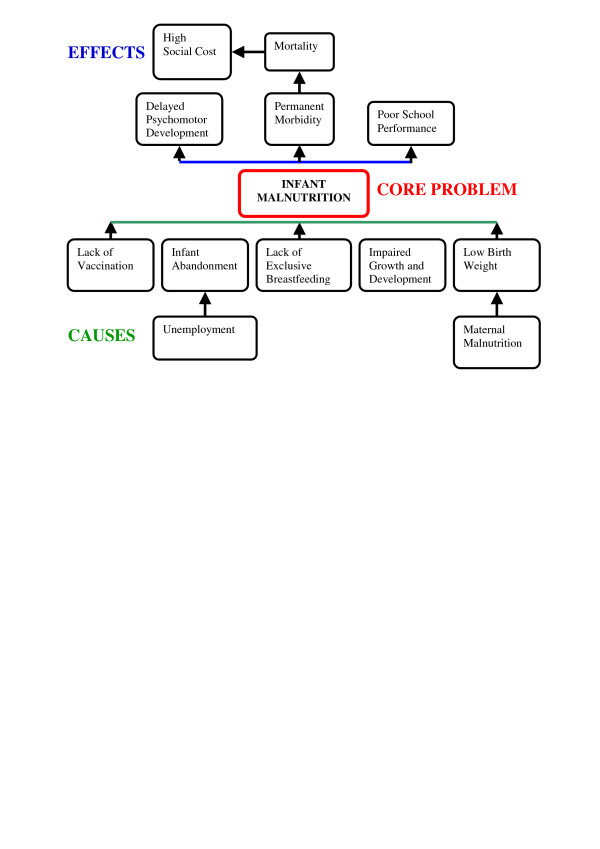
Problem tree for infant malnutrition from Participatory Workshop 1, Iquitos, Peru, April 1, 2005.

The top six priority problems for which cause and effect problem trees were to be developed were decided to be: 1) infant malnutrition; 2) adolescent pregnancy; 3) acute diarrhoeal illness/parasites/anaemia; 4) lack of basic sanitation; 5) low level of education and 6) STDs. As diarrhoea, parasites and anaemia are interrelated and have many common causes and effects, they were combined into one priority problem to maximize the information obtained from the participatory exercise. The problem tree for infant malnutrition which was developed by workshop participants is found in Figure 2. Proximate causes of infant malnutrition included lack of vaccination, infant abandonment (due to unemployment), lack of exclusive breastfeeding, impaired growth and development and low birth weight (due to maternal malnutrition). Effects of infant malnutrition included delayed psychomotor development, poor school performance and permanent morbidity leading to mortality and high social cost. Causes and effects of the other priority problems are listed in Table 2 and were similar to those for infant malnutrition.

**Table 2:** Proximate factors related to priority problems identified at Participatory Workshop 1, Iquitos, Peru, April 1, 2005.

**Priority Problem**		**Health Factors**	**Nutrition Factors**	**Education Factors**	**Environmental Factors**	**Social Factors**
**1. Infant malnutrition**	**CAUSES**	No vaccination; poor growth/development; low birth weight	Non-exclusive breastfeeding, poor food habits/quality			Infant abandonment
	**EFFECTS**	Morbidity; mortality; delayed psychomotor development		Poor school performance		High social cost
**2. Adolescent pregnancy**	**CAUSES**	Drug/alcohol abuse		Low education level		Sexual abuse; peer pressure; lack of family support
	**EFFECTS**	Abortion	Infant malnutrition	Dropping out		Low self-esteem; infant abandonment
**3. Acute Diarrhoeal Illness**	**CAUSES**	Intestinal parasites; poor hygiene habits	Non-exclusive breastfeeding		Environmental contamination; lack of basic sanitation	
	**EFFECTS**	Dehydration; death	Malnutrition			
**4. Lack basic sanitation**	**CAUSES**			Low education level	Open drainage; presence of waste; lack of sewer system	Rapid development/urbanization
	**EFFECTS**	Source of infection/contamination			Contamination of water supply; vector presence (rodents, insects)	
**5. Low level of education**	**CAUSES**		Malnutrition	Dropping out; repetition of grade		Gender inequality; economic inaccessibility
	**EFFECTS**	Drug abuse				Violence; low quality of life; poverty; low SE development;
**6. Sexually Transmitted Diseases**	**CAUSES**			Insufficient sexual education		Promiscuity; prostitution; risk taking; drug/alcohol abuse
	**EFFECTS**	Morbidity; mortality				Low quality of life

### Workshop 2

The second workshop, "Taller 2: Un Enfoque Multidisciplinario para Reducir la Pobreza Extrema" was held at the El Dorado Plaza Hotel in Iquitos, Peru on March 25, 2006. All previous participants who attended the first workshop were invited to attend. In addition, representatives of the Social Health Insurance Program (ESSALUD) and the regional government of Loreto were invited. There were a total of 45 participants, of which 21 (46.7%) were women.

Morning presentations by project investigators included results from the first workshop, school and household surveys and anticipated future research activities to be conducted in Belen. For the afternoon session, potential interventions were developed for priority problems including infant malnutrition, adolescent pregnancy, low level of education and STDs. Specific details of the interventions included intervention targets, components of interventions and barriers to implementation of the intervention (Table 3). Common barriers to implementation of interventions for all priority problems included cultural beliefs, low education level, and other social issues. Educational interventions were thought to be essential to overcome these barriers. Workshop participants expressed the overall benefits of targeting interventions to infant malnutrition to be: 1) improved psychomotor development; 2) decreased social cost; 3) decreased morbidity and mortality; and 4) improved school performance.

**Table 3:** Potential interventions for infant malnutrition from Participatory Workshop 2, Iquitos, Peru, March 31, 2005.

**Intervention Target**	**Components of Intervention**	**Barriers**
1. Complete immunization schedule	Health promotion	Migration
	Follow-up visits	Parental beliefs (e.g. adverse effects of vaccination)
2. Exclusive breastfeeding	Health promotion	Working mothers
	Prenatal control and follow-up visits	Parental beliefs
3. Adolescent family planning	Specific consult hours/location	Embarrassment
	Health education	Contraception access
4. Growth and Development programs	Follow-up visits	Cultural beliefs
	Health promotion	Migration
5. Nutritional behaviours	Education (food preparation, nutritional content, supplementation)	Lack of knowledge
		Cultural beliefs

One of the key intervention points for decreasing infant malnutrition was thought to be the promotion of exclusive breastfeeding; therefore, participants provided specific ways of targeting breastfeeding which included: 1) using a multi-sectoral approach for the promotion of exclusive breastfeeding; 2) strengthening family planning policy; 3) promoting responsible parenting; 3) having severe penalties for child exploitation; 4) strengthening Integrative Management of Childhood Illnesses program; 5) creating infant shelters; 6) decreasing infant abandonment; and 7) promoting exclusive breastfeeding to 6 months. According to participants, barriers to the promotion of exclusive breastfeeding included: 1) lack of support in public and private institutions for breastfeeding promotion; 2) lack of family planning; 3) neglect of vulnerable populations by government; 4) male chauvinism; 5) difficult mother-child contact (e.g. during working hours); 6) working conditions; and 7) cultural beliefs and misconceptions.

## Discussion

### Workshop Results

Based on results from the first workshop, the priority problems, as well as their associated causes and effects, fit into five general categories: 1) health; 2) nutrition; 3) education; 4) environment; and 5) social. Poverty was an underlying cause as well as a common effect of each problem. This is consistent with the cyclical nature of poverty as both a cause and effect of adverse health, nutrition and education outcomes. In addition, it was clear from the problem trees that many of the problems were interrelated with one another and were multiaetiological. For example, infant malnutrition was closely related to adolescent pregnancy, diarrhoea, lack of basic sanitation and low level of education. Many of the priority problems and related factors expressed in the first workshop, including an emphasis on targeting infant malnutrition, were confirmed by our survey results [10,11] as well as research that had previously been conducted in other areas of Peru [13].

In the second workshop, the participation of representatives from diverse sectors led to the identification of multidisciplinary interventions for the priority problems. Most interventions aimed to modify existing programs and infrastructure to better reach vulnerable populations. In addition, health promotion was a key component of most interventions and thought to be essential to overcome many of the cultural, physical, and educational barriers that would prevent effective implementation of interventions. Incorporating these features should improve the success of any interventions implemented in this community [13].

### Participatory Research

A participatory focus is one that is advocated by WHO, World Bank and others [14,15], including the Ministry of Health in Peru [16], and which allows for the involvement of important local stakeholders with different expertise and interests in all stages of the project. At both participatory workshops, there was a high attendance and participation rate from important local sectors in government, non-government, community and academic organizations, and national institutes. Employing a participatory research approach was thought to be the ideal way to empower local stakeholders and increase local ownership and therefore sustainability of this and future projects in Belen. Participants were able to contribute essential knowledge and understanding of the community, including political, economic, cultural and other Belen-specific factors in all stages of the project. Workshop participants, particularly the Ministry of Education, the Municipality of Belen and the Parent's Association, were also involved in and facilitated the design and implementation of the surveys to ensure that the most important information was obtained using locally-appropriate methodology. The involvement of local stakeholders in the surveys led to an increased acceptance of the research team by the community and a high participation rate of community members in the surveys. By involving representatives from important sectors in Belen in the first workshop and subsequent surveys, we were able to obtain a more accurate, complete and current picture of the priority health, education, nutrition and other related problems in Belen to improve the identification of appropriate interventions in the second workshop.

All of the problems that were identified as priority issues in the first workshop, and subsequent interventions that were identified in the second workshop, require coordination from multiple sectors in Belen; however, until now, most sectors worked independently of one another. The participatory approach increased multidisciplinary collaboration among representatives from diverse sectors by providing them with an opportunity to learn from and work with one another on common issues. This also led to increased capacity-building among participants by introducing them to new participatory techniques as well as knowledge of the role of other sectors in Belen.

Challenges to the participatory approach in this study included: 1) identifying appropriate representatives from the diverse sectors for participation at the workshop, particularly community representatives; 2) ensuring a balance in representation from the diverse sectors; 3) ensuring a high attendance rate from all participants, particularly in the afternoon participatory sessions; and 4) incorporating different or competing interests in the project.

Despite these challenges, the use of participatory research methods and the involvement of stakeholders in all stages of this project will improve the sustainability and impact of future interventions in Belen and ultimately contribute to improved health, nutrition, education and other related outcomes for the population.

## Conclusion

The two participatory workshops provided the first opportunity for important stakeholders from different sectors in Belen to convene together to speak about Belen-specific issues. The participatory approach used in this study will lead to increased sustainability and effectiveness of future multidisciplinary interventions, which will help to reduce both health and social inequalities in this community. In addition, the congruence between the knowledge of workshop participants, the results of the school and household surveys and the published literature will allow for the development of interventions that are both culturally and scientifically appropriate.

Based on the priority problems that were established in the two workshops and with data obtained from the school and household surveys, a five-year research program funded by the Canadian Institutes of Health Research is currently underway in the area. Its ultimate goal is to reduce poverty and health inequalities through targeted health, nutrition and education interventions. This program also aims to enhance local research capacity and to continue with its multidisciplinary and participatory approach.

## Competing interests

The author(s) declare that they have no competing interests.

## Authors' contributions

MC participated in study design, organizing and facilitating the workshop and revising the manuscript. SAJ contributed to the study design, organizing the workshop and manuscript preparation and revision. TWG participated in study design, organizing the workshop and manuscript preparation and revision. All authors have read and approved the final manuscript.
